# Meaning and lived experience of Iranian COVID‐19 survivors: A phenomenological study

**DOI:** 10.1002/brb3.3359

**Published:** 2024-01-02

**Authors:** Sedigheh Hasani‐Moghadam, Abou Ali Vedadhir, Fatemeh Alijani, Marzieh Azizi, Soghra Khani

**Affiliations:** ^1^ Department of Reproductive Health and Midwifery, Nasibeh Nursing and Midwifery School Mazandaran University of Medical Sciences Sari Iran; ^2^ Department of Population Health Sciences, Bristol Medical School University of Bristol Bristol UK; ^3^ Faculty of Social Sciences, Department of Anthropology University of Tehran Tehran Iran; ^4^ Sexual and Reproductive Health Research Center Mazandaran University of Medical Sciences Sari Iran; ^5^ Department of Reproductive Health and Midwifery, Sexual and Reproductive Health Research Center Mazandaran University of Medical Sciences Sari Iran

**Keywords:** COVID‐19 survivors, interpretative phenomenological analysis, Iran, qualitative study

## Abstract

**Background:**

The Coronavirus (COVID‐19) is among the most contagious diseases worldwide. During the first peak of the illness, COVID‐19 was considered a considerable crisis for survivors. This qualitative study explored the meaning and lived experience of Iranian COVID‐19 survivors. This qualitative study was conducted in Iran sometime after the onset of the coronavirus in 2020.

**Methods:**

This interpretative phenomenological analysis (IPA) was performed on twenty survivors of COVID‐19 disease, recruited through the purposeful sampling method via in‐depth semistructured interviews. The interviews were recorded and transcribed, and several codes were extracted. Data were analyzed using the MAXQDA software (v. 12).

**Results:**

The main themes and subthemes obtained from the data analysis included (1) *Taboo and stigma*: COVID‐19 as a monster, feelings of social exclusion and loneliness, an obvious sign of shamelessness and maltreatment, (2) *God's predestination*: God's will and test, COVID‐19 as a wake‐up call to remind low human power, (3) *Shadow of death*: The fear of death after positive test results, death is closer than the jugular vein, the mourning of a loved one's death, and mourning for an untimely death, (4) *Caregivers as an angel*: Family as an unrepentant supportive, know the level of family love and attention, and (5) *Rebirth and new life*: understand the higher value of health and pay more attention to self‐care behavior, and God gives us a golden chance to experience a better life.

**Conclusions:**

According to the results of this study, COVID‐19 survivors experience various issues regarding the nature of the disease, coping with the illness, and their social and psychological status affected by COVID‐19. Considering the multidimensional supportive programs, increasing public awareness and changing negative attitudes toward the patients and survivors of the pandemic for better rehabilitation and adjustment is essential.

## Graphical abstract

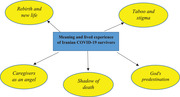

## 1 INTRODUCTION

Coronavirus disease (COVID‐19) is among the most contagious diseases (Habibzadeh & Stoneman, 2020), so it has even been declared a global public health emergency of international concern by the World Health Organization due to its high prevalence rate (Khosravi, 2021). This condition is closely related to severe acute respiratory syndrome and has been responsible for the outbreak since 2019. The emerging disease was first reported in December 2019 from Wuhan, the capital of Hubei Province in China (Peeri et al., 2020). The list of COVID‐19 symptoms is long, and their severity is linked to variants; its clinical symptoms are fever, cough, and shortness of breath, which can cause pneumonia, acute respiratory distress syndrome (ARDS), and kidney failure and can be transmitted through contaminated respiratory droplets (Lai et al., 2020). More fatalities have thus occurred in older patients or those with underlying diseases such as cardiovascular disease, diabetes mellitus, chronic lung disease, hypertension, and cancer (Khan et al., 2020)

According to the Ministry of Health and Medical Education, the total number of deaths from this disease has also increased in this country; this condition brings much anxiety, modifying individuals’ quality of life (Zhu et al., 2020). Factors such as human‐to‐human transmission and the high prevalence rate of this disease are accompanied by social and psychological risks (Beheshtkhoo et al., 2020), including patients’ fears of social consequences and the burden of this condition, such as stigmatization or labeling (Amiri & Vedadhir, 2018).

The literature review showed various studies were performed regarding the experience of COVID‐19 patients (Ardakani et al., 2022; Firouzkouhi et al., 2023; Realino et al., 2023; Sahoo et al., 2020). In this regard, a qualitative study in Iran showed that patients declared that during the disease, they experienced incredible clinical symptoms, fear of stigma, and bad memories of hospitalization (Firouzkouhi et al., 2023). Another study in Iran indicated that fear of spreading the disease to the family and others and also the experience of loneliness and distance from the family were the main concerns of the patients. In general, the patients quarantined at home during treatment experienced more favorable conditions than those admitted to the hospital. Some of the participants stated inappropriate behavior of some members of the healthcare providers to their illness (Amini et al., 2022). The results of a study that explored the lived experience of COVID‐19 survivors in Philippine isolation centers showed that COVID‐19 survivors had suffered more from the consequences of separation and discrimination than the disease's physiological effects (Romulo & Urbano, 2023). According to the literature, infected patients might face not only the physical effects of the disease but also its shocking consequences (Simbayi et al., 2007). At the onset of a disease, stigma may also mean that these people are labeled, stereotyped, discriminated against, and treated in isolation. This may lead to a loss of social status due to a perceived association with a particular disease. Such isolated treatments can adversely affect people with the disease, caregivers, family, relatives, friends, and their social interactions (Rewerska‐Juśko & Rejdak, 2022).

Although the literature review showed various qualitative studies were conducted regarding the lived experience of COVID‐19 patients or survivors, these survivors have multiple different occasions, and reporting them can be a good resource for recognizing the main challenging issues during disease and a considerable background for designing interventions in this regard through quantitative studies. In addition, assessing the results of other studies with our study showed some new themes that showed the importance of analyzing in this regard, so this study aimed to explore the meaning and lived experience of Iranian COVID‐19 survivors.

## 2 METHODS

### 2.1 Design and participants

This qualitative study, using an interpretative phenomenological analysis (IPA) (Larkin et al., 2021) and based on the Corec checklist for qualitative studies (Speziale et al., 2011), was conducted on a total number of twenty survivors of COVID‐19, living in the city of Sari, Mazandaran Province, Iran, after the onset of the coronavirus in 2020.

Participants were recruited through purposeful sampling without considering their gender and marital status. The inclusion criteria were the evidence of confirmed COVID‐19 infection (namely, positive polymerase chain reaction [PCR] test results or computerized tomography [CT] scan images of the lungs), Iranian nationality, professionals in the Persian language, have completed their quarantine period and willingness to participate in the study.

The participants were selected from different job statuses, such as healthcare providers of an educational hospital in Sari, Mazandaran province, and academic environments such as Nursing and midwifery school. In addition, houseworkers affected by COVID‐19 were selected by other participants of this study who knew them and invited them for interviews.

### 2.2 Data collection procedure and interview questions

The data collection tools included a sociodemographic characteristics questionnaire and in‐depth phenomenological semistructured interviews. The question guide was also prepared using the related literature and consultations with experts, and the final revisions were made after several pilot interviews.

Some of the questions in the interview guide were as follows:

The question, “Tell us about your perceptions and experiences of COVID‐19?” was asked as a guide. According to the study participants’ answers, exploratory inquiries, such as (a) elucidate your perception and experience of fear of others from contacting you during infection, (b) express your understanding and experience of being labeled as a COVID‐19 patient by others, (c) explain your meaning and lived experience of affecting by COVID‐19, and tell us about the way you cope with social and psychological pressures of infection, were addressed.

The researchers also observed the survivors’ nonverbal actions and reactions. They attempted to identify and understand the types and the quality of experiences they had during the disease and took notes to use during data analysis.

All the interviews were conducted in quiet and private locations selected by the participants for a maximum of 45 min. Notably, all the interviews were conducted by an experienced and familiar interviewer to the qualitative study. A second interview was performed to verify the data and bridge the possible gaps if required. All the interviews were correspondingly transcribed and coded. Notably, the interview sessions were continued until data saturation was reached, and authors emerged no new codes from the later interviews. For more certainty, three interviews were also conducted after finalizing the results. In addition to recording the interviews, field notes and observations were also employed to collect the data.

### 2.3 Ethical considerations

This study was approved with the research code of 7472 and the ethics code of IR.MAZUMS.REC.1399.7472, at the Sexual and Reproductive Health Research Center affiliated with Mazandaran University of Medical Sciences, Mazandaran, Iran. The researchers started the interviews with the eligible COVID‐19 survivors, meeting the inclusion criteria, upon obtaining permission from the Vice Chancellor's Office for Research and Technology at Mazandaran University of Medical Sciences, Mazandaran, Iran.

The research team explained the research methods and objectives and obtained informed consent to participate. Additionally, the samples’ information was obtained concerning their confidentiality.

### 2.4 The rigor of data

To increase the accuracy and robustness of the data, the criteria of credibility, transferability, conformability, and dependability were evaluated (Morse, 2015). To assess the data's credibility, the data analysis results were presented to other research team members, and their additional and critical comments were used. The transcribed interviews and the extracted themes were also given to the interviewees to check and match them with their experiences. To boost the transferability of the data, the appropriate samples were selected, that is, those who met the inclusion criteria for the interviews. To confirm the findings, two experts working in qualitative research and reproductive health were also asked to review the reports and transcripts and communicate their findings to determine the degree of similarities in the conclusions. To increase the dependability of the data, they were given to external researchers with experience in qualitative studies and blinded to the research to determine if they obtained the same results.

### 2.5 Data analysis

The data management was performed by the MAXQDA software v.12. The IPA was also employed to analyze the data in this qualitative research. After the complete transcription of the interviews, the researchers repeatedly reviewed and actively judged each interview. Then, the interviews were studied line‐by‐line, and the main points were identified. The themes characterizing each main topic were correspondingly determined, and a label or a title was assigned. In the next stage, the themes were placed in various categories based on their links, and each category was named. In this way, secondary themes were generated. Finally, the researchers explored the common patterns among the themes extracted and the categories formed from different interviews to find the dominating theme and how the theme could explain the expressions of the participants, leading to the formation of the main themes (Larkin et al., 2021).

## 3 RESULTS

This study conducted twenty interviews with Iranian COVID‐19 survivors aged 20–60. Their educational level also ranged from an undergraduate to an MD degree, and they were mostly married. Tables 1 and 2, respectively, outline the sociodemographic characteristics of the participants and lists the themes and subthemes extracted from the interviews. The qualitative data from the participant's interviews were placed into five themes after data analysis, as explained below.

**TABLE 1:** Sociodemographic characteristics of participants of the study.

#	Gender	Age	Occupation	Marital status	Education	Place of living	Self‐reported socioeconomic status (Vieira et al., 2022)
1	Male	40	Employee	Married	National diploma	Urban	Middle
2	Male	42	Employee	Married	Academic degree	Urban	Middle
3	Female	20	University student	Single	University student	Urban	Middle
4	Male	27	Self‐employed	Married	National diploma	Urban	High/upper
5	Female	25	Homemaker	Married	National diploma	Urban	Middle
6	Female	44	Psychiatrist	Married	Specialist/physician	Urban	High/upper
7	Female	55	Homemaker	Married	National diploma	Urban	High/upper
8	Male	60	Employee	Married	National diploma	Urban	Middle
9	Male	50	Employee	Married	Bachelor's degree	Urban	Middle
10	Male	40	Employee	Married	National diploma	Urban	Middle
11	Female	43	Employee	Married	PhD degree	Urban	Middle
12	Female	52	Homemaker	Married	Bachelor's degree	Urban	Middle
13	Male	29	Self‐employed	Married	Bachelor's degree	Urban	High/upper
14	Female	33	Employee	Married	Master's degree	Urban	High/upper
15	Male	45	Employee	Married	Academic degree	Urban	Middle
16	Male	28	Self‐employed	Married	Bachelor's degree	Urban	High/upper
17	Female	42	Employee	Married	Master's degree	Urban	High/upper
18	Female	25	Homemaker	Married	Master's degree	Urban	High/upper
19	Male	34	Self‐employed	Married	Bachelor's degree	Urban	High/upper
20	Female	46	Employee	Married	Bachelor's degree	Urban	Middle

**TABLE 2:** Themes, subthemes, and codes.

Theme	Subthemes	Codes
**Taboo and stigma**	COVID‐19 as a monster	A tough battle with viral bullets A cruel killer Bad shocks A terrible and incurable condition A pitiful and hopeless look at a person considered overburdening, annoying, and excessive
	Feelings of social exclusion and loneliness	Desperately lonely Separated from everyone and everything Feeling like a burden and destroyed The futility of doing work, wasting services, and eventually releasing them Grief, frustration, and tragedy
	An obvious sign of shamelessness and maltreatment	Family disintegration Fatal psychological blows from friends and loved ones (waiting for the patient to die) Loss of priority and value, along with minimum benefits Feelings of weariness and emptiness Pretending to be healthy (to maintain honor and dignity and even life and body)
**Predestination**	God's will God's test	Surrender and satisfaction for God's pleasure Being in the circle of divine decisions COVID‐19 as a part of life Achieving awareness, cognition, and management of one's thoughts
	COVID‐19 as a wake‐up call to remind low human power	Overcoming COVID‐19 as an obstacle that costs life A disturbing event sleeps and casts an ominous shadow
**Shadow of death**	The fear and phobia of death after positive test results	Fear of death Fear of the horrible name of the disease Fear of exposure to the virus Fear of the incurable nature of the virus
	Death is closer than the jugular vein.	The high possibility of death after infection Waiting for death after a positive result of the test
	The mourning of loved ones’ death	Mourning for the family member's death The fear of losing loved persons
	Mourning for an untimely death	Mourning of losing the living chance Mourning for being unlucky in life
**Caregivers as an angel**	Family as an unrepentant supportive	Having supportive family Comprehensive family care during the disease Risking one's life to care for during disease
	Know the level of family love and attention	The sacrifice of the family in care The extreme concern of the family about health status
**Rebirth and new life**	Understand the higher value of health	Increased perceived life value More attention to the chance of life after surviving
	More attention to self‐care behavior	Closer attention to health status after surviving Follow the self‐care program after surviving
	God gives us a golden chance to experience a better life	Thank God for rebirth after an incurable disease The appreciation of God for providing the golden chance for life The opportunity to experience a new life after surviving

### 3.1 Theme 1: Taboo and stigma

#### 3.1.1 COVID‐19 as a monster

In this study, COVID‐19 was a simile to a monster and the worst crisis of the COVID‐19 survivor's life, so it put them between the two paths of life and death, and they had to fight hard to stay alive. In this regard, a participant said:

- “*As far as this, I have never been in such a life‐threatening situation that you do not know what will happen and whether you will survive or not. I did not think that coronavirus would do this to me*” (Female, 25 years old, married).

Another survivor declared that:

- *COVID‐19 was like a terrible nightmare and a disappointing life event. The thought of whether I will survive scares me more than the symptoms of the disease. I was lucky to survive this cruel killer and monster virus* (Female, 42 years old, married).

Another participant said:

- “*Others viewed me as a monster after I got the virus. They think that COVID‐19 patients are offenders and even an additional burden, and they must die*” (Male, 29 years old, married).

#### 3.1.2 Feelings of social exclusion and loneliness

Feeling lonely and experiencing social isolation results from COVID‐19, particularly at the onset of the diagnosis, and approximately all COVID‐19 survivors stated the fear of others having face‐to‐face contact with patients. In this regard, a participant declared that:

- “*I thought I was excluded from society, and everyone was running away from me*” (Male, 27 years old, married).

Another COVID‐19 survivor stated:

- “*I felt isolated. I thought everything was over. I needed to stay somewhere lonely until I die*” (Female, 33 years old, married).

The feelings of loneliness and social isolation resulted from the COVID‐19 patients’ thoughts about being away from others. A young man said:

- “*I felt isolated. I felt that everything was over. There was no life, and then I had to stay somewhere to die*” (Male, 28 years old, married).

The COVID‐19 patients did not refer to health care centers, as much as possible, due to their worries about undesirable confrontations and refusals to provide services or to receive incomplete ones, even if they were facing problems. A participant said:

- “*When I came to the emergency room and went to a place for COVID‐19 patients to have my injection, they looked at me in a way that I did not want to come once again to the hospital even though I needed to see the doctor*” (Female, 55 years old, married).

#### 3.1.3 An obvious sign of shamelessness and maltreatment

Several COVID‐19 patients stated that, upon reporting their condition to their workplace, they had witnessed inappropriate treatment by the authorities and their work, and their financial and social status had been endangered. A participant represented that:

- “*Once I informed the authorities of the positive test results, they mistreated me and said they would deduct my salary*” (Male, 45 years old, married).

Some COVID‐19 survivors had only informed their family members, and their relatives did not know about the disease. For instance

- “*I suffer if others know about my condition. They may maltreat me and my family, and we may lose face*” (Female, 20 years old, single).

The participants' experience in some studies indicated that disclosing the disease could lead most healthcare workers (HCWs) to refuse to provide health care for such patients. Most nurses were also afraid of injections. They even feared approaching the patients. The patients even complained about the discrimination between them and other non‐COVID‐19 cases. As an example

- “*I was maltreated by some doctors. When I went to have an injection, they had a terrible attitude, looked at me unsympathetically, and blamed me*” (Female, 20 years old, single).

### 3.2 Theme 2: God's predestination

#### 3.2.1 God's will and test

Some patients considered COVID‐19 as providence from God. A participant said in this regard:

- “*You cannot fight with your destiny, and we must be satisfied with whatever the will of God is*” (Female, 20 years old, single).

According to some patients, COVID‐19 was considered a divine decision. In this regard, a woman said:

- “*This was a divine decision and our destiny. I think COVID‐19 was a kind of divine test, and now it has been our destiny*” (Female, 55 years old, married).

#### 3.2.2 COVID‐19 as a wake‐up call to remind low human power

COVID‐19 as a disease has so far had a significant impact on the human lifestyle, including spiritual revival and getting closer to God. A young woman declared in this regard:

- “*COVID‐19 could wake me up as a flip to know myself and make my relationship with God and the saints better*” (Female, 25 years old, married).

Regarding the fatal nature of the disease and the low power of humans to overcome this disease, a middle‐aged woman stated:

- *While struggling with the disease to survive, I realized how weak humans are and should not be too proud of themselves. COVID‐19 reminded me of this* (Female, 46 years old, married).

Spirituality was the main issue for patients to become calm and withstand terrible conditions. In this regard, a man said:

- *I always thought that I was decisive and that the impossible was meaningless. COVID‐19 considerably proved to me that I was weak and I was waiting for a miracle from God*. *This disease reminded me again that no power but the power of God can survive us from difficult situations* (Male, 28 years old, married)

Another participant who was a physician said:

- *I feel weak and angry when I see that I am a physician but have no control over the disease stages. Everything is out of my control. In front of your eyes, your close family members are suffering from such a terrible disease, but as a physician, you can do nothing* (Female, 44 years old, married).

### 3.3 Theme 3: Shadow of death

One of the central experiences of COVID‐19 survivors was the shadow of death after the positive results of the COVID‐19 test.

#### 3.3.1 The fear and phobia of death after positive test results

The fear of death was the biggest concern of the COVID‐19‐positive cases.

In this regard, a participant said:

- *For several days, I felt symptoms similar to COVID‐19, but I thought that I had these symptoms because of fear. At my husband's insistence, we went and took the COVID‐19 test. When I saw the test's positive result, I panicked, felt shortness of breath, and the whole world spun around me. I was looking at my test result sheet bewildered* (Female, 43 years old, married).

Some patients knew about the high probability of death caused by this disease. Stress might arise once the respiratory symptoms become more severe in people, so they are very anxious and constantly think about death. For example

- “*Thinking of being buried in a dig smaller than several meters, under lime, was torturing me*” (Female, 55 years old, married).

The COVID‐19 patients were not satisfied with the burial and the funeral ceremony. For instance,

- “*God forbid that I die so that I have no funeral and no one comes to my ceremony*” (Female, 52 years old, married).

#### 3.3.2 Death is closer than the jugular vein

As this virus's mortality rate and lethality were so high, most participants declared that they were waiting for the death time. In this regard, a woman stated:

- *When my test result was positive, I said that I would die, and I was waiting for my time to die, and I thought that death was closer to me. It was a terrible feeling that a virus would destroy me after all these years* (Female, 46 years, married).

Another participant said:

- “*I felt isolated. I felt that everything was over. There was no life, and then I had to stay somewhere to die*” (Male, 28 years old, married).

Some COVID‐19 survivors pictured the disease as full of sorrow and stress for the future. A man declared that:

- “*Every morning, I waited to die. No one came to see me. Their words were mere compliments to help me, and I used to say ‘no’ and ‘do not bother.’ They used to accept them easily. I cried in the mornings and felt like I was dying at night*” (Male, 29 years old, married).

#### 3.3.3 The mourning of loved ones' death

The news of the family member's death was one of the terrible and incredible events during the disease and pandemic. In this regard, a man said

- “*Before me, my wife was infected with COVID‐19 and was hospitalized in the intensive care unit. I was also infected, but I was quarantined at home. I was worried about my wife. I asked others about her condition by phone. Lately, whenever I called, no one answered correctly. I doubted it until I was informed of her death a few days later. The whole world collapsed on me”* (Male, 50 years old, married).

Another participant said:

- “*My husband's mother died due to being infected with the COVID‐19 virus. I feel very guilty thinking that maybe I was a vector when I was in contact with her, and I transferred the virus to her”* (Female, 55 years old, married).

#### 3.3.4 Mourning for an untimely death

Many participants stated that one of the main issues during their disease was psychological distress regarding the high possibility of death. In this regard, a participant said:

- “*Each second of my disease, I was awaiting exposure to death. I continuously thought about my senses in that terrible time and grieved for them beforehand*” (Female, 33 years old, married).

### 3.4 Theme 4: Caregivers as an angel

#### 3.4.1 Family as an unrepentant supportive

It is apparent that caregivers have a vital role during the COVID‐19 pandemic, and approximately all participants believed they received comprehensive multidimensional support. In this regard, a participant said:

- “*My family is always by my side in any situation; their existence is like a miracle. I didn't even think they would get the disease from me when I got infected. They inspired me, and I once again proved that they are the angels of my life*” (Female, 20 years old, single).

#### 3.4.2 Know the level of family love and attention

“*My husband and I were infected with COVID‐19 at the same time. We were quarantined in the same room. You may not believe that even though my husband had much more lung involvement than me, he worried about me and tried to take care of me despite his unsuitable condition. I realized again how much he loved me*” (Female, 52 years old, married).

“*During my illness, I realized that no one could be more compassionate and take care of me than my family*” (Female, 33 years old, married).

### 3.5 Theme 5: Rebirth and new life

#### 3.5.1 Understand the higher value of health

The COVID‐19 survivors believed they felt rebirth and God gave them a new life, so they should appreciate it. A participant declared that:

- *After overcoming that monstrous and unbelievable disease and escaping death, I now understand more than anyone else the value of health God has given us. I take care of it however I can* (Male, 27 years old, married).

#### 3.5.2 More attention to the self‐care behavior

Affected by COVID‐19 was a considerable alarm for attention to self‐care behavior. In this regard, a participant stated that:

- *Even though I had COVID‐19 symptoms, I was not convinced to go and take a coronavirus test. When I went to take the test, more than 70% of the time was involved. Maybe I would have gone earlier; I would not have suffered so much. After surviving, I promised to constantly follow up on my health* (Male, 45 years old, married).

#### 3.5.3 God gives us a golden chance to experience a better life

Approximately all of the survivors thank God for continuing their lives after being affected by that horrible disease. A survivor declared that:

- *When I saw that many patients with the same condition as I had died, but I survived, I felt God gave me another golden opportunity to appreciate and live a better life* (Female, 43 years old, married).

Another participant said:

- *Life is never as precious to me as it is now that I have survived a terrible disease, and I am very grateful to God for the opportunity to live again* (Female, 44 years old, married).

## 4 DISCUSSION

This qualitative study explored the meaning and lived experience of Iranian COVID‐19 survivors. The results of this study led to the extraction of 5 themes and 13 subthemes. Overall, survivors of COVID‐19 faced intensified challenges regarding their experiences reported in this study.

Taboo and stigma were among the first themes extracted by the interview. Stigma is a phenomenon by which a person is discredited or disgraced by society and is then excluded. Also, stigmatization is associated with chronic and incurable diseases and their modes of transmission, which are related to individuals’ behaviors, especially those that do not comply with social norms (Amiri & Vedadhir, 2018). Overall, due to the destructive nature of the disease, having positive results on the COVID‐19 test, especially in the first peak of the disease, was considered a stigma, so many people tried to deny their condition.

In this regard, a survey showed that labeling patients could lead to their exclusion from society and subsequently hiding the disease, which could have destructive effects on public health (Faghankhani et al., 2022). In qualitative research on stigma in Iran, two themes, external concepts (the feelings of blame, shame, and discrimination) and internal ideas (namely, social exclusion, self‐denial, and disappointment), had been similarly presented. In the domain of ​​blame and shame, the patients had experienced blameful looks, misjudgment, labeling, exploration of the disease, and avoidant behaviors. In addition, in the domain of ​​discrimination, the patients suffered from the HCWs’ cautious behaviors, lack of treatment, unemployment, and dismissal. Concerning the sense of self‐denial, the patients had also demonstrated behaviors such as refusal to refer to health care centers, disease concealment, and feelings of loneliness and isolation, accompanied by frustration in life, no marriage, waiting to die, and suicide (Rahmati et al., 2020). The results of a study in South Korea that explored the experience of COVID‐19 survivors showed that the participants experienced social stigma and feelings of guilt, a sense of negative attitudes of society toward patients induced by social networks and media, which negatively influenced their psychosocial function (Son et al., 2021). Therefore, the essential interventions can be developed by better understanding the destructive effects of stigma and discrimination, minimizing stigmatization, and improving general policies aimed at raising public awareness through mass media and social networks, the exchange of information, and changes in HCWs’ attitudes (Logie et al., 2013).

In the present study, the interviews showed that the COVID‐19 survivors analogized this condition to a monster, limiting social activities, employment, and so on. To justify this analogy, the naming and framing model, presented first by Viktor Smith, could be employed (Smith, 2021). This model deals with understanding words' power across disciplines, domains, and methods. The power that comments have not only to objects and phenomena perceived in reality but also to those known and exploited by humans to make others aware of their presence and to shape their perceptions (Smith, 2021). This model consisted of four levels. Levels 1–3 are all related to naming, namely, “giving something a name.” While Level 1 means naming something, level 2 denotes how it is called, and Level 3 suggests how the underlying and original signs shape the full communication potential of the name. In contrast, Level 4 means naming, that is, “calling something by a specific name or a complete set of names,” thus affecting people's perceptions of everything being spoken without necessarily creating new names (Smith, 2021). A study in Turkey on the effect of labeling multiple sclerosis on health values showed that labeling could cause a condition, significantly reducing health status and quality of life (Green et al., 2017).

To the authors’ knowledge, no study has been conducted on naming and framing COVID‐19. However, the results of some studies highlight the necessity of correct management of this disease from structural, political, and media perspectives so that people's negative attitudes change toward positive ones, and ultimately, stigma and discrimination are minimized (Dhanani & Franz, 2021; Jo & Chang, 2020).

This study also demonstrated that the COVID‐19 survivors considered this condition God's will, God's test, and an alarming sign or a wake‐up call to remind human power. The survey revealed that stronger religious beliefs could reduce mortality, stress, excitement, depression, illness, suicide, and addiction. This indicates that the mental health dimension should be considered as much as the physical one during health care services (Azizani kolvizi, 2016). The results of a study also introduced reliance on God as a divine example, that is, those entrusted to the divine power could overcome problems, including COVID‐19 (Ismaili & Heydari, 2021). Consistent with the results of this study, a survey reported that a critical situation such as the COVID‐19 infection could be the basis of spirituality, strengthen the remembrance of God and trust in him, and give meaning to life through religion and rethinking death (Asadi et al., 2023). According to these findings, it can be concluded that faith in and remembering God are the factors for gaining peace of mind, especially during the COVID‐19 pandemic. It can also be claimed that problems such as diseases cause a kind of human awakening, and human beings can feel safe or protected through prayers and supplications (Ismaili & Heydari, 2021).

In this study, survivors believed that when affected by COVID‐19, they felt their death was near, and they needed a miracle to survive. Overall, the fatal nature of this virus induced these stressful feelings among patients. In this regard, the results of a qualitative showed that factors such as the incredible death statistics worldwide, the exacerbated physical exhaustion and symptoms, limited, defined drug protocol for treatment, and the low successful treatment rate lead to many patients affected by COVID‐19 equal to death and this issue negatively affect their psychological well‐being. False broadcasted information through social media and repeated news regarding the nature of disease and death plays an essential role in creating anxiety and fear among participants (Silwal et al., 2021). Another qualitative study showed that the feeling of being close to death is one of the most essential negative experiences of patients affected by COVID‐19. The participants declared that announcements of deaths on social media made them concerned regarding the incurability of the disease (Ardakani et al., 2022). Several tragic deaths might accompany life. Bereavement and grief represent the mental reactions of the survivors of a significant loss (Khosravi, 2021). The expression “bereavement” stands for a universal experience of failure or defeat, especially after the death of a loved one (Peeri et al., 2020). Grief is also a cognitive process that requires confronting and restructuring thoughts about the deceased, the experience of loss, and the altered world in which the survivor must now live. Accordingly, the psychological effects of pandemics have always been the focus of attention among researchers (Duan & Zhu, 2020; Lima et al., 2020). During the emerging COVID‐19 pandemic in Wuhan, China, in late 2019, such psychological problems also became evident (Zarghami, 2020). Regarding the mourning experiences of families of dead infected with COVID‐19, families and friends cannot contact their loved ones and express their love, support, and sympathy. They alone cope with grief, sorrow, and emotional exhaustion (Mojarad et al., 2021).

One of the main extracted themes in this study was the essential role of caregivers during the disease, so approximately all participants mentioned the supportive role of their families during their illness. Social support, especially from friends, diagnosis duration, and higher education levels, could also be associated with low stigma (Abachi & Behravan, 2020; Galvan et al., 2008). The results of a qualitative study in China revealed that a lack of social support for the affected person could be accompanied by adverse effects such as anxiety, depression, grief, a sense of guilt, isolation, reduced life expectancy, further restriction in social networks, unemployment, loss of income, and misunderstanding of social interactions (Abachi & Behravan, 2020). A study showed that family support was a significant motivation for a successful recovery, overcoming difficulties, and rebuilding their lives with their family's help (Kim & Park, 2021). It can be argued that interpersonal relationships decline due to the need for social distancing and the quarantine of the infected cases, and this causes a sense of distance and social isolation and, ultimately, a feeling of rejection and worthlessness. Hence, it seems that psychological measures should be taken for this purpose.

Another theme from the interview was the sense of rebirth and the chance for new life. The results of studies that explored the lived experiences of COVID‐19 survivors showed that healing from incurable and horrific diseases led to more understanding of the values of life, experiencing pleasurable situations, and feeling reliving and reborn among participants (Firouzkouhi et al., 2023; Nygaard et al., 2020). Consistent with our study results, a study showed that COVID‐19 patients declared that they pay more attention to their health status, seek medical care, and follow health information‐seeking behavior after surviving (Santiago‐Rodriguez et al., 2021).

### 4.1 Limitations of this study

The limitation of this study was only a limited number of patients who recovered from COVID‐19 participated in the study. Also, most of the survivors had no experience of hospitalization, so the experiences of patients hospitalized in the intensive care unit need to be investigated.

## 5 CONCLUSIONS

It seems that the results of this study have helped us better understand the lived experience of COVID‐19 survivors. According to the results of this study, COVID‐19 survivors experience various issues regarding the nature of the disease, coping with the illness, and their social and psychological status affected by COVID‐19. In addition, the patients’ experiences in this study can be applied to identifying the perceived needs of patients and their caregivers regarding their treatment and care by HCWs. Accordingly, it is suggested to raise public awareness and enhance HCWs’ knowledge to remove stigma and discrimination against patients by providing general education through the media and social networks. Besides, it is recommended to eliminate stigma and discrimination against patients and survivors by encouraging support groups and authorities.

## AUTHOR CONTRIBUTIONS

**Sedigheh Hasani‐Moghadam**: conceptualization; writing—original draft. **Abou Ali Vedadhir**: data curation; methodology. **Fatemeh Alijani**: data curation; methodology. **Marzieh Azizi**: writing—review and editing. **Soghra Khani**: data curation; methodology.

## CONFLICT OF INTEREST STATEMENT

The authors declared no conflict of interest in this study.

### PEER REVIEW

The peer review history for this article is available at https://publons.com/publon/10.1002/brb3.3359.

## Data Availability

The datasets generated during and/or analyzed during the current study are available from the corresponding author upon reasonable request.
